# Child–Pugh grade deterioration stratified by the etiology after transcatheter arterial chemoembolization as initial treatment for hepatocellular carcinoma

**DOI:** 10.1038/s41598-024-53709-6

**Published:** 2024-02-14

**Authors:** Kengo Yoshitomi, Tsuguru Hayashi, Shinji Oe, Michihiko Shibata, Yuichi Honma, Masaru Harada, Yohei Kooka

**Affiliations:** 1 Third Department of Internal Medicine, School of Medicine, University of Occupational and Environmental Health, Kitakyushu, Japan; 2 Department of Hepatology, Sendai Kousei Hospital, 4-15 Hirosemachi, Aoba-Ku, Sendai, 980-0873 Japan

**Keywords:** Hepatocellular carcinoma, Liver function, Child Pugh grade, Gastroenterology, Liver cancer, Liver cirrhosis

## Abstract

Transcatheter arterial chemoembolization (TACE) is a standard treatment for intermediate-stage hepatocellular carcinoma (HCC). However, TACE can cause deterioration of liver functions. We aimed to identify the factors that influence deterioration of liver function after TACE. We retrospectively analyzed 262 patients who underwent TACE as initial treatment for HCC with Child–Pugh grade A. We divided them into three groups stratified by the etiology of underlying liver disease. Patients were classified into hepatitis B virus (HBV) group, hepatitis C virus (HCV) group, and non-HBV / non-HCV (NBNC) group. Liver functions at one month after TACE and time to Child–Pugh grade B or C were compared between the three groups. The HBV, HCV and NBNC groups contained 23, 123 and 116 patients, respectively. The decline in albumin level after TACE was significantly higher in NBNC group than other groups (p = 0.02). NBNC group showed a shorter time to Child–Pugh grade deterioration compared with HBV group and HCV group (p < 0.001). Multivariate Cox regression analysis showed that NBNC group was a significant factor for Child–Pugh grade deterioration (Hazard ratio 3.74, 95% confidence interval 1.89–7.40, p < 0.001). These results revealed that liver functions worsened most remarkably in NBNC group after TACE.

## Introduction

Hepatocellular carcinoma (HCC) is one of the most common tumors and a leading cause of cancer related-death^1^. HCC has a high recurrence rate and liver functions tend to deteriorate with each repeated treatment^2–5^. Deterioration of liver functions can limit treatment options in HCC treatment strategy, leading to a poorer prognosis^6,7^. Therefore, tumor conditions and liver functions should be considered in selecting treatment methods when HCC occurs^8,9^.

Transcatheter arterial chemoembolization (TACE) is a standard therapy that has been used to treat HCC for many years. TACE is recommended for unresectable HCC. TACE has shown to be most effective when the number and size of tumors were limited^10^. However, TACE deteriorates liver functions^11,12^. When the number or size of tumors is large, chemoembolization from more central hepatic arteries (non-selective TACE) has to be performed and liver functions tend to deteriorate.

In the past few years, molecular targeted agents (MTA) and immune checkpoints inhibitors (ICI) have emerged and have prolonged survival in some patients with HCC^13–18^. However, we can use these drugs only when liver functions preserved. Therefore, it is important to discuss whether TACE or systemic therapy (MTA and ICI) should be the initial treatment in Child–Pugh grade A^19^. The Asia Pacific Primary Liver Cancer Expert (APPLE) consensus statement established that not all patients benefit from TACE in terms of tumor burden and liver functions^20^. Cases in which no anti-tumor effect could be expected from TACE or in which liver function is likely to deteriorate were defined as unsuitable for TACE, and systemic therapy should be given priority. To predict the HCC patients who are unsuitable for TACE, the up to 7 criteria and modified albumin-bilirubin (mALBI) grade are considered^21,22^. Without up to 7 or image findings that predict poor prognosis are known to predict antitumor efficacy^23–25^. Also, without up to 7 and mALBI grade 2b are considered as criteria likely to worsen liver functions. However, no further conditions have been studied to identify patients who are unsuitable for TACE from the point of liver function and the etiology of underlying liver disease.

In this study, clinical question of this research is whether the etiology of liver disease influences Child–Pugh grade deterioration. We investigated factors that influences Child–Pugh grade deterioration after the initial TACE, focusing particularly on the etiology of liver disease. We also studied the aggravation of liver function after a single TACE treatment, as well as prognosis, stratified by etiology.

## Results

### Patient characteristics

We evaluated 262 patients with HCC whose initial treatment was TACE. The number of patients with the HBV group, the HCV group and the NBNC group were 23, 123 and 116, respectively. In the HBV group, 20 patients (87.0%) began taking nucleotide analogue at least after the initial treatment for HCC. In the HCV group, 101 patients (82.1%) had not achieved sustained virologic response (SVR) at the time of last observation. Of the patients who achieved SVR, 45.4% were treated by IFN-free direct acting antivirals. Sixteen patients (13.0%) achieved SVR before hepatocarcinogenesis and six patients (4.9%) achieved SVR after hepatocarcinogenesis. The baseline characteristics are shown in Table 1. Age, BMI and the rate of DM were significantly higher and the muscle volume was significantly lower in the NBNC group. The proportion of male and prothrombin time were significantly higher in the HBV group. However, Child–Pugh score and ALBI score did not differ among 3 groups. Tumor factors also showed no significant differences among the 3 groups.

**Table 1:** Baseline characteristics in hepatocellular carcinoma patients who underwent TACE as initial treatment.

	HBV group (N = 23)	HCV group (N = 123)	NBNC group (N = 116)	p-value
Age (IQR), years	67 (60–72)	75 (68–80)	78 (71–82)	< 0.001
Male, %	87.0	67.5	79.3	0.045
Ethanol consumption, g/day	14 (0–78)	0 (0–66)	36 (0–90)	0.051
Presence of DM, %	26.1	33.3	50.0	0.001
BMI (IQR), kg/m^2^	22.1 (20.5–24.2)	21.9 (20.2–24.6)	23.4 (21.7–25.8)	0.002
Muscle volume (IQR)	6.0 (4.9–7.0)	4.6 (3.9–5.6)	4.0 (3.4–5.0)	< 0.001
Albumin (IQR), g/dL	3.8 (3.6–4.2)	3.9 (3.7–4.1)	3.8 (3.6–4.1)	0.694
Bilirubin (IQR), mg/dL	0.7 (0.5–0.8)	0.6 (0.5–0.9)	0.7 (0.5–1.0)	0.509
Prothrombin time (IQR), %	90.6 (81.0–100.0)	84.2 (76.7–93.4)	89.6 (79.8–100.0)	0.012
AFP (IQR), ng/mL	16.0 (6.6–57.1)	18.1 (6.4–149.1)	8.8 (4.6–202.7)	0.239
DCP (IQR), mAU/mL	254.0 (41.8–696.3)	187.0 (30.0–2125.0)	385.0 (57.3–4854.8)	0.176
Child–Pugh score, 5 points, %	60.9	68.3	67.2	0.771
ALBI score	− 2.59 (− 2.89 to − 2.27)	− 2.61 (− 2.84 to − 2.42)	− 2.55 (− 2.77 to − 2.34)	0.250
BCLC stage 0/A/B/C	1/7/12/3	15/31/64/13	4/28/65/19	0.214
Within up to 7, %	56.5	69.9	56.9	0.091

*AFP* alfa-fetoprotein, *ALBI* albumin-bilirubin, *BCLC* Barcelona Clinic Liver Cancer, *BMI* body mass index, *CI* confidence interval, *DCP* des-gamma-carboxy prothrombin, *DM* diabetes mellitus, *HBV* hepatitis B virus, *HCV* hepatitis C virus, *HR* hazard ratio, *PT* prothrombin time, *TACE* transcatheter arterial chemoembolization.

### Outcome of initial TACE

Of the 262 patients, 240 patients received radiological evaluations. For 22 cases, contrast enhanced CT or contrast enhanced MRI were not taken due to renal function problems, but plain CT and tumor markers were considered for the next treatment. The overall rates of CR, PR, SD and PD were 38.7%, 34.6%, 11.3% and 15.4%, respectively. Within up to 7, the rates of CR, PR, SD and PD were 55.2%, 27.9%, 5.2% and 11.7%, respectively. Without up to 7, the rates of CR, PR, SD and PD were 9.3%, 46.5%, 22.1% and 22.1%, respectively. Stratified by the etiology of liver disease, the rates of CR, PR, SD and PD in HBV group were 30.4%, 52.2%, 8.7% and 8.7%, respectively. In the HCV group, the rates of CR, PR, SD and PD were 47.4%, 28.9%, 16.7% and 7.0%, respectively. In the NBNNC group, the rates of CR, PR, SD and PD were 31.1%, 36.9%, 15.5% and 16.5%, respectively. The difference of the 3 groups were not significant (p = 0.054).

### Change of liver functions at 1 month after the initial TACE

One month after initial TACE, albumin and prothrombin time were decreased in all groups. The average level of albumin, bilirubin and prothrombin time decreased 0.27 g/dL, 0.03 mg/dL and 2.9%, respectively. Compared by the etiology of the liver disease, the NBNC group showed a significant decrease in albumin (p = 0.021) (Table 2). Bilirubin and prothrombin time showed no significant differences, when stratified by the etiology. In linear regression analysis in change of albumin after the initial TACE, univariate analysis showed that des-gamma-carboxy prothrombin (DCP) (estimated regression coefficient: − 0.19, 95% CI − 0.28 to − 0.09, p < 0.001), NBNC group (estimated regression coefficient: − 0.13, 95% confidence interval (CI) − 0.23 to − 0.04, p = 0.006), within up to 7 (estimated regression coefficient: − 0.20, 95% CI − 0.29 to − 0.10, p < 0.001) were significant factors (Table 3). The multivariate analysis found that DCP (estimated regression coefficient: − 0.12, 95% CI − 0.22 to − 0.02, p = 0.025), NBNC group (estimated regression coefficient: − 0.11, 95% confidence interval (CI) − 0.20 to − 0.02, p = 0.023) and within up to 7 (estimated regression coefficient: − 0.12, 95% CI − 0.23 to − 0.02, p = 0.024) were the significant factors.

**Table 2:** Change of liver functions at 1 month after initial TACE.

	HBV group	HCV group	NBNC group	p-value
Albumin, g/dL	− 0.1	− 0.2	− 0.3	0.021
Bilirubin, mg/dL	0	0	0	0.069
Prothrombin time, %	− 1.5	− 1.7	− 3.0	0.185

*HBV* hepatitis B virus, *HCV* hepatitis C virus, *TACE* transcatheter arterial chemoembolization.

**Table 3:** Linear regression analysis of changes in albumin after initial TACE.

	Univariate analysis			Multivariate analysis		
	Estimated regression coefficient	95% CI	p-value	Estimated regression coefficient	95% CI	p-value
Age (> 75 years)	− 0.03	− 0.13–0.06	0.513			
Male	− 0.04	− 0.15–0.07	0.433			
Ethanol consumption	− 0.07	− 0.18–0.03	0.172			
Presence of DM	− 0.08	− 0.18–0.02	0.100			
BMI (> 25.0 kg/m^2^)	0.05	− 0.06–0.16	0.336			
Presence of sarcopenia	− 0.10	− 0.23–0.01	0.071			
AFP (> 400 ng/mL)	− 0.06	− 0.17–0.06	0.334			
DCP (> 400 mAU/mL)	− 0.19	− 0.28 to − 0.09	< 0.001	− 0.12	− 0.22 to − 0.02	0.025
Child–Pugh score (6)	0.03	− 0.07–0.13	0.569			
Etiology (NBNC)	− 0.13	− 0.23 to − 0.04	0.006	− 0.11	− 0.20 to − 0.02	0.023
ALBI grade (2)	0.07	− 0.02–0.17	0.131			
Within up to 7	− 0.20	− 0.29 to − 0.10	< 0.001	− 0.12	− 0.23 to − 0.02	0.024
Responder of TACE	0.02	− 0.08–0.13	0.670			

*AFP* alfa-fetoprotein, *ALBI* albumin-bilirubin, *CI* confidence interval, *DCP* des-gamma-carboxy prothrombin, *DM* diabetes mellitus, *HBV* hepatitis B virus, *HCV* hepatitis C virus, *PT* prothrombin time, *TACE* transcatheter arterial chemoembolization.

### Time to Child–Pugh deterioration after the initial TACE

**Figure 1:**
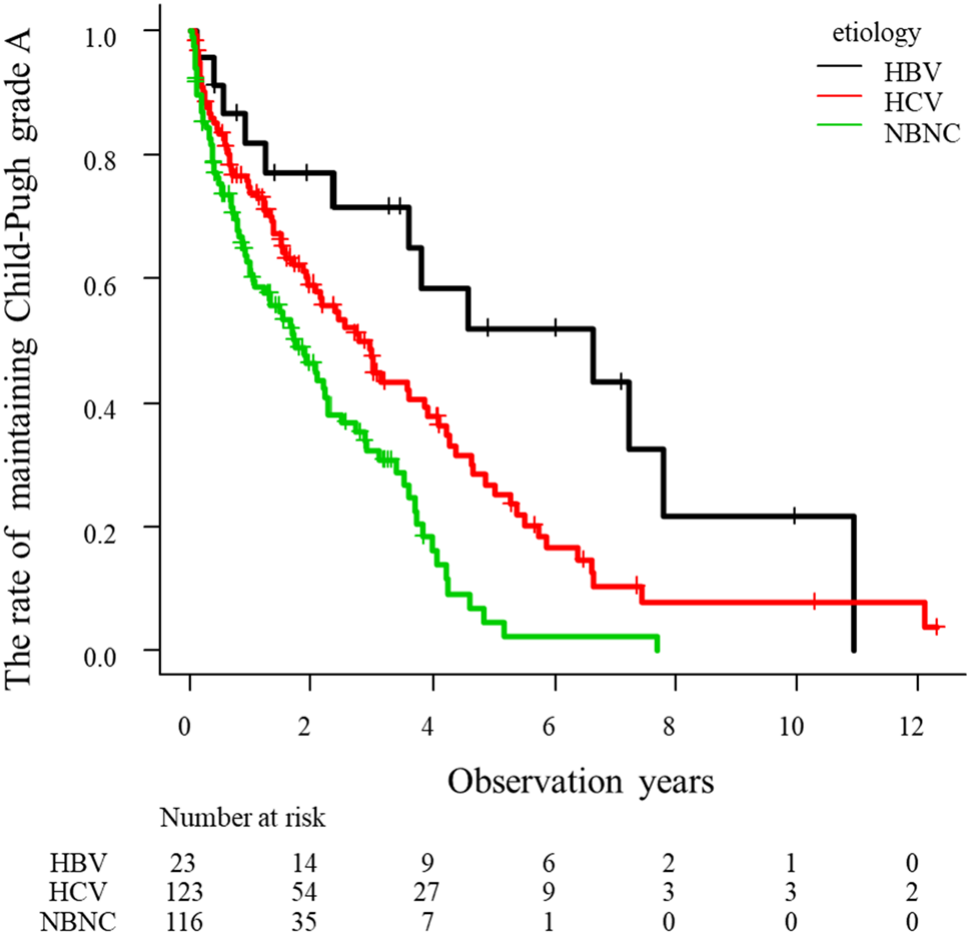
HYPERLINK "sps:id::fig1||locator::gr1||MediaObject::0"The rate of maintaining Child–Pugh grade A stratified by etiology in HCC patients who underwent TACE as the initial treatment. The NBNC group showed shorter time to Child–Pugh grade deterioration compared with the HBV group (p < 0.001) and HCV group (p < 0.001).

**Figure 2:**
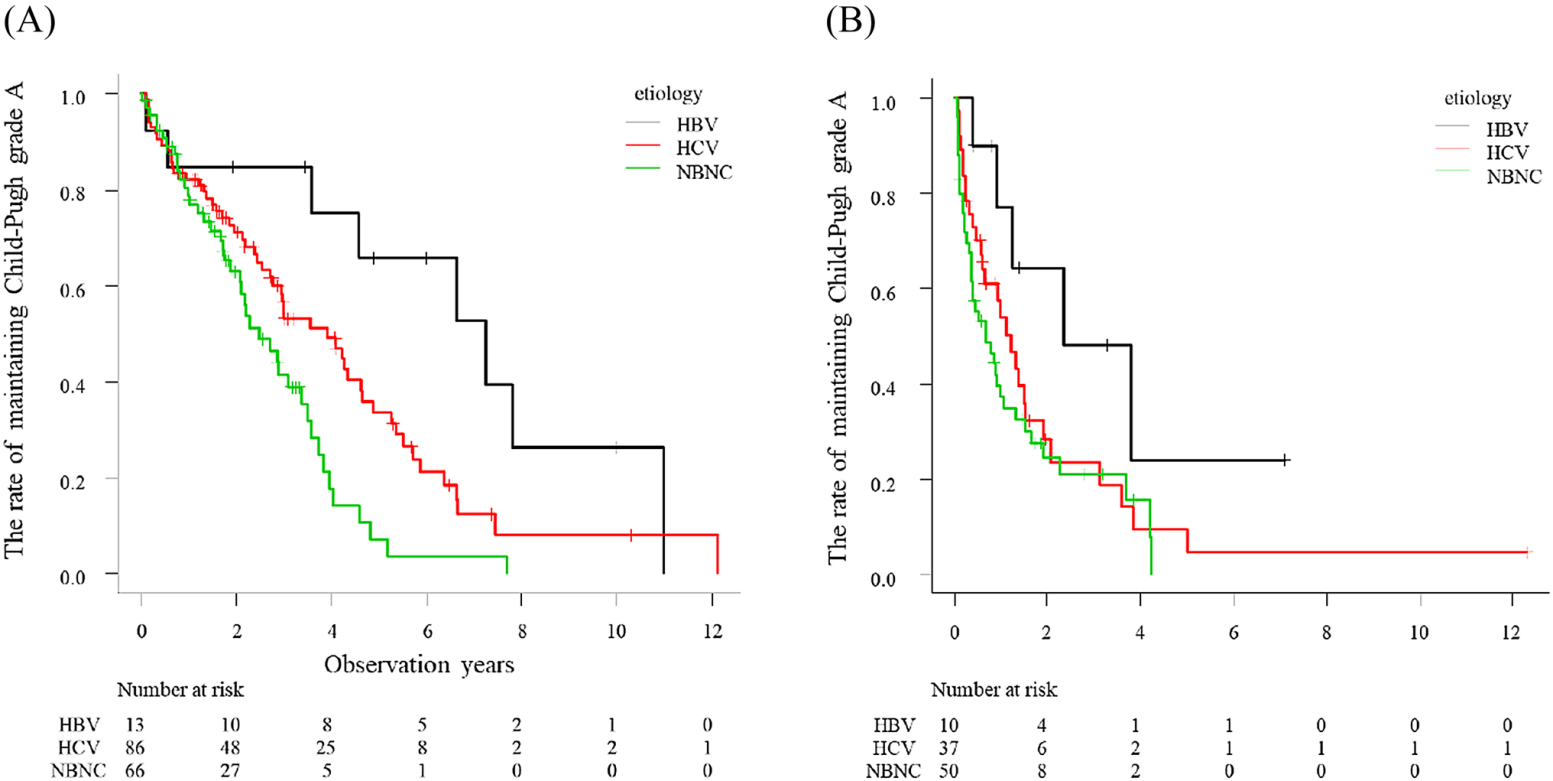
The rate of maintaining Child–Pugh grade A stratified by the etiology when divided by up to 7 criteria. (**A**) Within up to 7, the NBNC group showed a significantly shorter time to Child–Pugh grade deterioration compared to the HBV group (p < 0.001) and HCV group (p = 0.008). (**B**) Without up to 7, the NBNC group showed a significantly shorter time to Child–Pugh deterioration only when compared to the HBV group (p = 0.03), and no difference compared with the HCV group (p = 0.35).

The median observational period after initial TACE was 2.0 years. The median time to Child–Pugh grade deterioration was 2.3 years. The cumulative Child–Pugh deterioration rate after initial TACE was 31.1%, 56.6% and 78.6% at 1, 3 and 5 years, respectively. Univariate analysis using the Cox proportional hazards model revealed that the factors associated with Child–Pugh grade deterioration were presence of sarcopenia (Hazard ratio (HR) 1.64, 95% CI 1.12–2.38, p = 0.010), alfa-fetoprotein (AFP) (HR 1.97, 95% CI 1.38–2.82, p < 0.001), DCP (HR 2.28, 95% CI 1.68–3.12, p < 0.001), Child–Pugh score 6 (HR 2.26, 95% CI 1.66–3.07, p < 0.001), NBNC group (HR 1.90, 95% CI 1.39–2.60, p < 0.001), ALBI grade 2 (HR 2.55, 95% CI 1.87–3.49, p < 0.001), within up to 7 (HR 2.41, 95% CI 1.77–3.29, p < 0.001) and responder (HR 0.39, 95% CI 0.27–0.54, p < 0.001) (Table 4). Multivariate analysis showed that Child–Pugh score 6 (HR 2.29, 95% CI 1.51–3.49, p < 0.001), NBNC group (HR 1.64, 95% CI 1.13–2.38, p = 0.009), ALBI grade 2 (HR 1.78, 95% CI 1.18–2.67, p = 0.006), within up to 7 (HR 1.89, 95% CI 1.28–2.79, p = 0.001) and responder (HR 0.45, 95% CI 0.30–0.68, p < 0.001) were significant independent factors influencing Child–Pugh grade deterioration. Stratified by the etiology, the NBNC group showed shorter time to Child–Pugh grade deterioration compared with the HBV group (p < 0.001) and HCV group (p < 0.001) (Fig. 1). When we divided the up to 7 criteria by group, the NBNC group showed a significantly shorter time to Child–Pugh grade deterioration than the HBV group (p < 0.001) and HCV group (p = 0.008) within up to 7 (Fig. 2A). However, among patients without up to 7, the NBNC group showed a significantly shorter time to deterioration than the HBV group (p = 0.03). There was no significant difference between the NBNC and HCV groups (p = 0.35) (Fig. 2B).

**Table 4:** Univariate and multivariate Cox regression analysis for Child–Pugh deterioration.

	Univariate analysis			Multivariate analysis		
	HR	95% CI	p-value	HR	95%CI	p-value
Age (> 75 years)	1.28	0.94–1.74	0.115			
Male	0.90	0.64–1.27	0.554			
Ethanol consumption (> 60 g/day)	1.35	0.98–1.86	0.070			
Presence of DM	1.06	0.79–1.43	0.699			
BMI (> 25.0 kg/m^2^)	0.88	0.63–1.22	0.435			
Presence of sarcopenia	1.64	1.12–2.38	0.010	1.32	0.86–2.02	0.199
AFP (> 400 ng/mL)	1.97	1.38–2.82	< 0.001	1.26	0.80–1.99	0.326
DCP (> 400 mAU/mL)	2.28	1.68–3.12	< 0.001	1.32	0.89–1.97	0.166
Child–Pugh score (6)	2.26	1.66–3.07	< 0.001	2.29	1.51–3.49	< 0.001
Etiology (NBNC)	1.90	1.39–2.60	< 0.001	1.64	1.13–2.38	0.009
ALBI grade (2)	2.55	1.87–3.49	< 0.001	1.78	1.18–2.67	0.006
Within up to 7	2.41	1.77–3.29	< 0.001	1.89	1.28–2.79	0.001
Responder of TACE	0.39	0.27–0.54	< 0.001	0.45	0.30–0.68	< 0.001

*AFP* alfa-fetoprotein, *ALBI* albumin-bilirubin, *CI* confidence interval, *DCP* des-gamma-carboxy prothrombin, *DM* diabetes mellitus, *HBV* hepatitis B virus, *HCV* hepatitis C virus, *HR* hazard ratio, *TACE* transcatheter arterial chemoembolization.

In the propensity score matching analysis, no significant differences were found in patients background factors between NBNC group and HBV and HCV group (Supplementary Table). The time to Child–Pugh grade deterioration in NBNC group was significantly shorter than HBV and HCV group (p = 0.022) (Supplementary Figure).

### Prognosis of HCC patients

**Figure 3:**
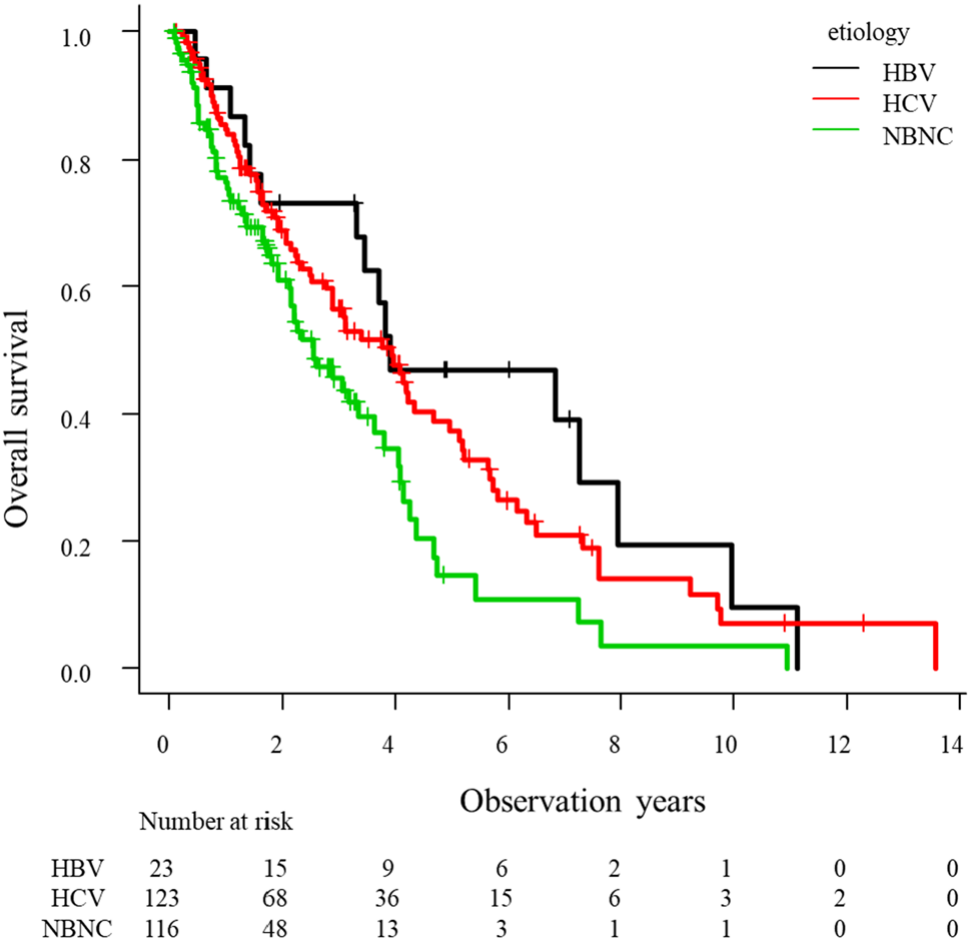
Overall survival of HCC patients who underwent TACE as initial treatment stratified by etiology. NBNC group showed a poorer prognosis than the HBV group (p = 0.01) and HCV group (p = 0.01).

The median survival time (MST) was 3.3 years in all patents. The cumulative death after initial TACE was 18.0%, 46.2% and 68.8% at 1, 3 and 5 years, respectively. Stratified by the etiology of the liver disease, NBNC group showed a poorer prognosis than the those of HBV group (p = 0.01) and HCV group (p = 0.01) (Fig. 3). Total number of HCC treatments was 5.0, 3.8 and 3.1 in the HBV, HCV and NBNC groups, respectively (p < 0.01). Number of HCC treatment during Child–Pugh grade A was 4.5, 3.2 and 2.6 in HBV, HCV and NBNC groups, respectively (p < 0.01). The Cox proportional hazards model revealed that the factors associated with overall survival were presence of sarcopenia (HR 1.80, 95% CI 1.20–2.69, p = 0.004), AFP (HR 2.21, 95% CI 1.51–3.25, p < 0.001), DCP (HR 2.90, 95% CI 2.10–4.02, p < 0.001), Child–Pugh score 6 (HR 1.81, 95% CI 1.31–2.51, p < 0.001), NBNC group (HR 1.61, 95% CI 1.16–2.23, p = 0.004), ALBI grade 2 (HR 1.92, 95% CI 1.40–2.65, p < 0.001), Within up to 7 (HR 2.60, 95% CI 1.87–3.61, p < 0.001) and responder (HR 0.31, 95% CI 0.22–0.44, p < 0.001) in univariate analysis (Table 5). Multivariate analysis showed DCP (HR 1.96, 95% CI 1.29–2.97, p = 0.001), Child–Pugh score 6 (HR 1.71, 95% CI 1.10–2.66, p = 0.017), Within up to 7 (HR 1.85, 95% CI 1.22–2.81, p = 0.004) and responder (HR 0.37, 95% CI 0.24–0.57, p < 0.001) were significant independent factors influencing overall survival.

**Table 5:** Univariate and multivariate Cox regression analysis for overall survival.

	Univariate analysis			Multivariate analysis		
	HR	95%CI	p-value	HR	95%CI	p-value
Age (> 75 years)	1.23	0.89–1.69	0.209			
Male	1.29	0.88–1.88	0.194			
Ethanol consumption (> 60 g/day)	1.35	0.95–1.91	0.098			
Presence of DM	0.96	0.69–1.31	0.777			
BMI (> 25.0 kg/m^2^)	0.65	0.45–0.93	0.020	0.92	0.59–1.43	0.716
Presence of sarcopenia	1.80	1.20–2.69	0.004	1.56	0.98–2.46	0.060
AFP (> 400 ng/mL)	2.21	1.51–3.25	< 0.001	0.98	0.60–1.61	0.948
DCP (> 400 mAU/mL)	2.90	2.10–4.02	< 0.001	1.96	1.29–2.97	0.001
Child–Pugh score (6)	1.81	1.31–2.51	< 0.001	1.71	1.10–2.66	0.017
Etiology (NBNC)	1.61	1.16–2.23	0.004	1.19	0.81–1.76	0.377
ALBI grade (2)	1.92	1.40–2.65	< 0.001	1.50	0.98–2.29	0.060
Within up to 7	2.60	1.87–3.61	< 0.001	1.85	1.22–2.81	0.004
Responder of TACE	0.31	0.22–0.44	< 0.001	0.37	0.24–0.57	< 0.001

*AFP* alfa-fetoprotein, *ALBI* albumin-bilirubin, *CI* confidence interval, *DCP* des-gamma-carboxy prothrombin, *DM* diabetes mellitus, *HBV* hepatitis B virus, *HCV* hepatitis C virus, *HR* hazard ratio, *TACE* transcatheter arterial chemoembolization.

## Discussion

We investigated whether the etiology of underlying liver disease influenced Child–Pugh grade deterioration after the treatment with TACE in HCC patients. The NBNC group showed a marked decrease in serum albumin level after the initial TACE. This could help explain why the NBNC group showed a shorter time from Child–Pugh grade A to B. Also, the NBNC group had the smallest number of HCC treatment times.

We think that these facts are clinically important in HCC treatment strategy. Recently, it is some debate as to whether TACE or systemic therapy (MTA and ICI) should be chosen when liver resection and radiofrequency ablation was impossible^26–29^. To better prioritize treatments, up to 7 criteria and ALBI grade have been reported as useful criteria for evaluating tumor and liver functions. Without up to 7 and ALBI grade 2b were more likely to show deterioration of liver function after TACE. However, our study found that the etiology of liver disease was a significant factor influencing Child–Pugh deterioration, independent of the up to 7 criteria and ALBI grade. Especially in the case of within up to 7, different treatment strategies stratified by the etiology of liver disease could be needed. When TACE was not able to achieve CR in one treatment, early introduction of systemic therapy before repeating TACE should be considered. This suggestion is supported by the fact that 44.8% of patients could not obtain CR even within up to 7. In fact, in lenvatinib-TACE sequential therapy that had a high CR and PR, NBNC group was most common in patients who enrolled in this study^30^. However, further study is needed to confirm our hypothesis that systemic therapy should be introduced early in NBNC patients.

Despite of the recent increases in non-viral HCC, many details remain unknown^31^. The carcinogenic process of HCC varies by etiology of underlying liver diseases. This is because intracellular signaling pathways differ depending on the etiology. Also, such differences could affect responsiveness to HCC treatment. Therefore, it has recently been pointed out that we need to focus on etiology when considering treatment strategy.

The reason why the NBNC group showed shorter time to Child–Pugh grade deterioration is unclear. However, we considered several possible reasons. First, decreased muscle volume could lead to deterioration of liver function. In our study, muscle volume was significantly low in the NBNC group, compared with HBV and HCV groups. This may be due to the high rate of heavy drinkers or patients with DM in the NBNC group. Ethanol and high blood glucose could cause muscle atrophy. Muscles are involved in the processing of ammonia^32^. Therefore, when muscle volume is reduced, blood ammonia level increases and consumption of branched chain amino acid increases. Also, ethanol increases the production of myostatin, a type of cytokine released by muscle. Myostatin inhibits protein synthesis in the muscle^30^. This amino acid imbalance leads to hypoalbuminemia. This suggests that loss of muscle volume in the NBNC group could affect the deterioration of liver function. In fact, it has been reported that HCC patients after TACE had a better prognosis after rehabilitation intervention^33,34^. In this study, the presence of sarcopenia was a significant factor for Child–Pugh deterioration in univariate analysis. However, sarcopenia was not significant in multivariate analysis. Therefore, we consider that other facts were involved.

Second, the rate of DM is higher in the NBNC group. In patients with DM, insulin resistance elevates. Insulin resistance induces inflammation and fibrosis in the liver. Also, insulin resistance associated with oxidative stress and endoplasmic reticulum stress. These facts could have induced deterioration of liver functions after TACE. Moreover, insulin resistance is an independent factor associated with HCC recurrences^35^. This is because hyperinsulinemia has been considered to be involved in occurrence and recurrence of HCC due to the mitogenic and proliferative effect of insulin^36^. This could be the reason for the low response rate of NBNC group to TACE in our study.

Third, different immune responses due to the etiology of liver disease could played a role. Recently basic and clinical studies have demonstrated that nonalcoholic steatohepatitis (NASH)-induced HCC has a different immunological response to ICI^37^. CD8^+^ T cells were found to be increased and activated in NASH. CD8^+^ T cells could promote the progression of NASH and be involved in the impairment of anti-tumor surveillance. Also, meta-analysis of three large randomized controlled studies for advanced HCC showed that immune therapy did not improved survival in patients with non-viral HCC^38^. A mechanism similar to these occurs after TACE. However, these three reasons on their own could not explain the difference in deterioration of liver functions due to the etiology of underlying liver disease.

We considered that the HBV group was able to maintain good liver function because HBV was controlled by nucleotide analogue. Most patients in HBV group were taking nucleotide analogue in this study. Antiviral therapy for HBV improved liver functions and prognosis of HCC patients^39^. However, in the HCV group, only 17.9% of patients achieved virologic control during the course of treatment. This difference could explain the difference in maintenance of liver function between the HBV group and HCV group^40^.

The reason why previous studies have not shown that the etiology influences liver functions could be due to patient background. In our study, the number of patients within up to 7 was relatively high. Also, Child–Pugh deterioration stratified by the etiology of liver disease was significantly faster in patients within up to 7. In a previous study, the number of patients within up to 7 is small^25^. This could lead to a significant difference in Child–Pugh deterioration by etiology.

Our study had some limitations. First, this was a retrospective observational study. A prospective randomized control study is needed. Especially, the HBV group was rather small in this study. Second, we have not further stratified the NBNC group. In the NBNC group, a wide variety of etiologies, including NASH, alcohol related/associated liver disease, autoimmune hepatitis and primary biliary cholangitis were enrolled. Therefore, it is necessary to examine the change of liver functions according to these etiologies within a large sample. Third, the amount of ethanol consumption after initial HCC treatment is unknown. We instruct HCC patients to stop drinking to improve prognosis. We believe that few patients continue to drink. However, the exact percentage is unknown.

In conclusion, when the etiology of liver disease in HCC patients is not HBV or HCV, liver function is likely to be aggravated by TACE. Therefore, we suggest that early introduction of systemic therapy before repeating TACE should be considered in patients with HCC of NBNC etiology.

## Methods

### Patients

This retrospective study enrolled consecutive patients with Child–Pugh grade A　unresectable HCC with who underwent TACE as the initial treatment for HCC at our hospital from May 2005 to January 2020. Patients with the following conditions were excluded from the study; (1) underwent radiofrequency ablation immediately after TACE and (2) incomplete data. The diagnosis of HCC was based on histopathological findings by liver biopsy or characteristic radiological findings, such as contrast enhanced computer tomography (CT) or contrast enhanced magnetic resonance imaging (MRI). Barcelona Clinic Liver Cancer (BCLC) stage was used for the evaluation of HCC staging^9,10^. Underlying liver diseases were categorized according to the presence of chronic hepatitis infection. Patients who were positive for hepatitis B surface antigen or hepatitis C antibody were classified into hepatitis B virus (HBV) or hepatitis C virus (HCV) group, respectively. Those who were negative for these factors were placed in non-HBV and non-HCV (NBNC) group. We used the psoas muscle area index to indicate muscle volume. The psoas muscle area index (psoas muscle area at the middle of the third lumbar vertebra (cm^2^)/height (m)^2^) was manually calculated from CT findings at diagnosis of HCC. The presence of sarcopenia was then judged by the cut off value proposed by Hamaguchi et al.^38^ AASLD guidelines for the treatment of HCC were used for the selection of initial treatment^10^. After initial TACE, patients underwent regular follow-up with measurement of blood samples and CT or MRI examinations every 1 to 3 months. When the therapeutic effect was insufficient, additional treatment was performed. HCC recurrence was diagnosed according to radiological findings and/or tumor markers. The treatments for HCC recurrence were selected according to tumor and liver condition. This study was approved by the institutional ethical board in accordance with the Declaration of Helsinki (H29-078). This study is a retrospective observational study. Therefore, the ethics committee decided that informed consent is not required and waived. The name of ethics committee is Ethics Committee of Medical Research, University of Occupational and Environmental Health, Japan. The receipt number is H29-078. We announced publicly that patients could refuse to participate in this study if patients desire.

### Transcatheter arterial chemoembolization

TACE was performed when curative treatments were impossible. Even if the size and number of the tumor were small, TACE was selected depending on the size, location and arterial supply of the tumor. For example, when the tumor was close to the blood vessels or the diaphragm, TACE was selected instead of radiofrequency ablation.

TACE was performed selectively, using a microcatheter. After the microcatheter was inserted into the target arterial branch, TACE was performed by one of 3 methods; conventional TACE (cTACE), drug eluting beads TACE (DEB-TACE) or bland TAE. In cTACE, an anticancer drug and iodized oil were injected followed by injection of gelatin sponge particles. The anticancer drugs included epirubicin, mitomycin, cisplatin and miriplatin. In DEB-TACE, embolization of tumor blood vessels was achieved using beads adsorbed with anticancer drugs. In bland TAE, embolization alone was performed without the use of anticancer drugs. At 1 month after TACE, patients underwent follow-up examination. Follow-up assessments included liver functions, tumor markers and contrast enhanced CT. We evaluated radiological response of TACE according to the modified Response Evaluation Criteria in Cancer of the Liver (mRECIST). Responders were defined as complete response (CR) or partial response (PR). Non responders were defined as stable disease (SD) or progressive disease (PD).

### Endpoint of this study

The primary end points of this study were Child–Pugh grade deterioration. Child–Pugh deterioration was the time from time of the first treatment to Child–Pugh grade B or C. We did not defined aggravation of liver functions immediately after TACE as Child–Pugh deterioration. The second end point was overall survival.

### Statistical analysis

All categorical variables were analyzed using χ^2^-test or Fisher’s exact test, and continuous variables were compared using Kruskal Wallis test. P values < 0.05 were considered statistically significant. We compared age, sex, body mass index (BMI), muscle volume, ethanol consumption, presence of diabetes mellitus (DM), liver functions and tumor factors at diagnosis of HCC, stratified according to the etiology. Ethanol consumption was shown as daily ethanol intake when at the time HCC was diagnosed. Child–Pugh grade deterioration and overall survival were evaluated by the Kaplan–Meier curve, and differences between the two groups were assessed using the log-rank test. A Cox proportional hazards model was tested to determine the factor associated with endpoints. The cut off values that were used in univariate and multivariate analyses were medians of all patients or values that were used previously^41^. All variables with p value < 0.05 from the univariate analyses were included in multivariate analyses. Continuous numeric variables were expressed as median and interquartile range (IQR). We also evaluated liver functions at 1 month after initial TACE. Changes in liver functions were compared according to the etiology. Linear regression analyses were performed to identify the factors associated with decreased albumin levels. Propensity score was estimated using a logistics regression model with age, presence of DM, BMI, presence of sarcopenia. We used propensity scores to carry out one to one nearest neighbor matching, within a caliper of 0.2. Propensity score matching was results in 144 patients (HBV and HCV group, N = 72, NBNC group, n = 72). All statistical analyses were performed using the Statistical Package for the Social Science (SPSS) version 25 (SPSS Inc., Chicago, IL, USA) and Easy R (EZR) version 1.29 (Saitama Medical center, Jichi Medical University, Saitama, Japan), a graphical use interface for R (The R Foundation for Statistical Computing, Vienna, Austria)^42^.

### Supplementary Information

Supplementary Information.

## Data Availability

The authors confirm that the data supporting the findings of this study are available within the articles.
