# Supplementary material for: Child–Pugh grade deterioration stratified by the etiology after transcatheter arterial chemoembolization as initial treatment for hepatocellular carcinoma
Source: Sci Rep. 2024 Feb 14;14:3707. doi: 10.1038/s41598-024-53709-6 (PMC10867004; doi:10.1038/s41598-024-53709-6)

|  | HBV and HCV group | NBNC group | p-value |
| --- | --- | --- | --- |
|  | (N=74) | (N=74) |  |
| Age (IQR), years | 76 (68-80) | 77 (69-81) | 0.768 |
| Male, % | 71.6 | 78.4 | 0.448 |
| Ethanol consumption, g/day | 14 (0-72) | 36 (0-89) | 0.189 |
| Presence of DM, % | 43.2 | 36.5 | 0.502 |
| BMI (IQR), kg/m^2^ | 22.7 (20.5-25.0) | 22.8 (21.0-24.8) | 0.672 |
| Presence of sarcopenia | 82.4 | 83.8 | 1.000 |
| Albumin (IQR), g/dL | 3.9 (3.6-4.1) | 3.9 (3.7-4.1) | 0.897 |
| Bilirubin (IQR), mg/dL | 0.7 (0.5-0.9) | 0.7 (0.5-1.0) | 0.739 |
| Prothrombin time (IQR), % | 85.6 (77.1-95.3) | 88.3 (80.1-100.0) | 0.100 |
| AFP (IQR), ng/mL | 11.6 (6.4-68.8) | 11.2 (4.8-869.2) | 0.942 |
| DCP (IQR), mAU/mL | 112 (30-2118) | 425 (67-2927) | 0.055 |
| Child-Pugh score, 5 points, % | 71.6 | 70.3 | 1.000 |
| ALBI score | -2.58 | -2.57 | 0.734 |
| Within up to 7, % | 71.6 | 59.5 | 0.166 |
| Responder of TACE | 79.7 | 68.2 | 0.169 |

**Supplementary table** Baseline characteristics in hepatocellular carcinoma patients who underwent TACE as initial treatment after propensity score matching. AFP, alfa-fetoprotein; ALBI, albumin-bilirubin; BMI, body mass index; CI, confidence interval; DCP, des-gamma-carboxy prothrombin; DM, diabetes mellitus; HBV, hepatitis B virus; HCV, hepatitis C virus; PT, prothrombin time; TACE, transcatheter arterial chemoembolization.

**Supplementary Figure**


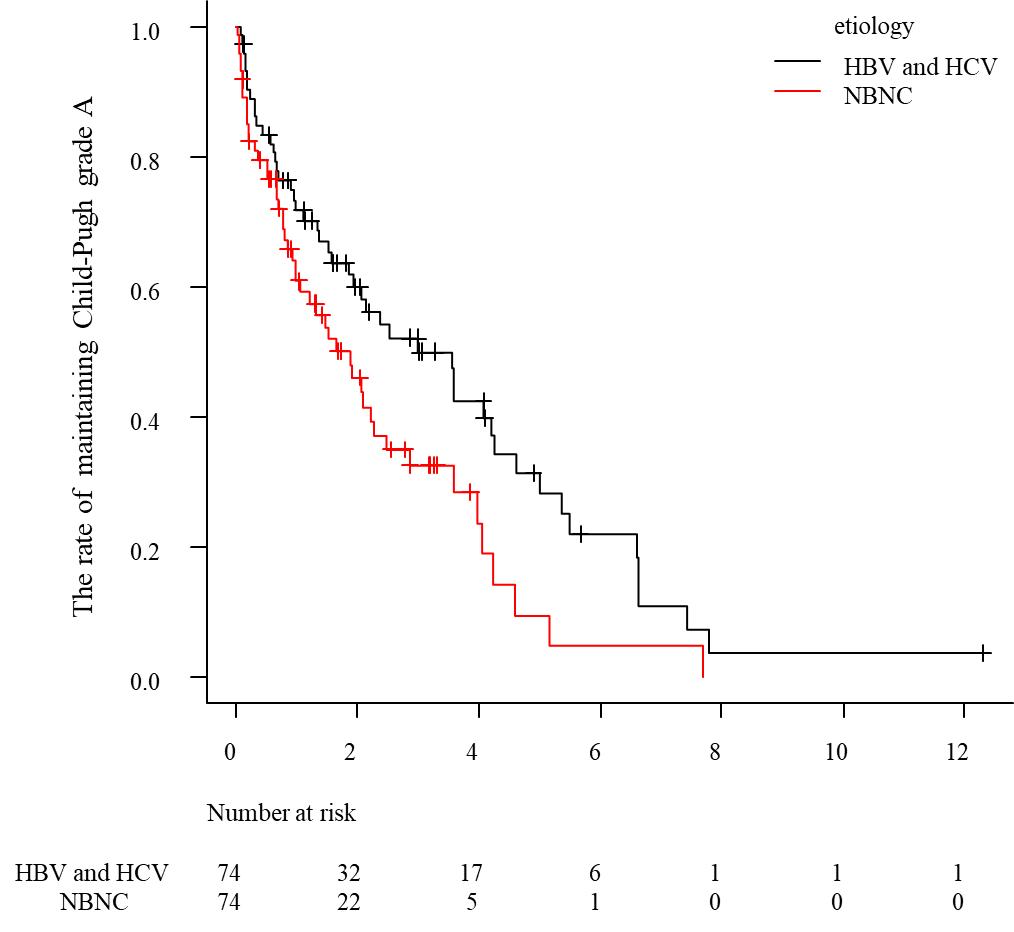

Supplement: Supplementary file 1 — Supplementary Information. [file 41598_2024_53709_MOESM1_ESM.docx]
